# Crystal structure and Hirshfeld surface analysis of chiral *catena*-poly[l-histidinediium [[diiodido­cuprate(I)]-μ-iodido] monohydrate]

**DOI:** 10.1107/S2056989025010023

**Published:** 2025-11-18

**Authors:** Valerii Y. Sirenko, Valeriia N. Ovdenko, Vadim A. Potaskalov, Mircea-Odin Apostu, Il’ya A. Gural’skiy

**Affiliations:** a Department of Chemistry, Taras Shevchenko National University of Kyiv, Volodymyrska St. 64/13, Kyiv 01601, Ukraine; b Department of General and Inorganic Chemistry, National Technical University of Ukraine "Igor Sikorsky Kyiv Polytechnic Institute", Beresteiskyi Pr. 37, 03056 Kyiv, Ukraine; c Department of Chemistry, Faculty of Chemistry, Al. I. Cuza University of Iasi, Carol I Blvd. 11, Iasi 700506, Romania; Harvard University, USA

**Keywords:** crystal structure, distortion indices, l-histidine, chirality, helical, Hirshfeld surface analysis, materials, one-dimensional halides, copper(I), amino acids, iodides, *A*_2_Cu*X*_3_-type compounds

## Abstract

The title compound, {(C_6_H_11_N_3_O_2_)[CuI_3_]·H_2_O}_*n*_ or (l-HisH_2_)CuI_3_·H_2_O (**1**), is a chiral organic–inorganic compound that crystallizes in the monoclinic *P*2_1_ space group. The asymmetric unit of **1** consists of one diprotonated l-histidinium cation {4-[(2*S*)-2-aza­niumyl-2-carb­oxy­eth­yl]-1*H*-imidazol-3-ium}, one Cu^+^ cation, three iodide anions, and one co-crystallized water mol­ecule. The Cu^+^ cations is four-coordinated by iodide anions forming a [CuI_4_] unit. Structural analysis of the [CuI_4_] unit using the Baur, τ_4_, and τ_4_′ indices reveals its slight deviation from ideal tetra­hedral geometry. Two iodide anions from each [CuI_4_] unit bridge adjacent Cu^+^ centers, forming chiral left-handed helical [CuI_3_]_*n*_^2*n*−^ polymeric chains. The biprotonated l-histidinium cations balance their negative charge and form N—H⋯I, O—H⋯I, and weak C—H⋯I hydrogen bonds with the [CuI_3_]_*n*_^2*n*−^ chains. According to the Hirshfeld surface analysis, the main contributions to the crystal packing arise from H⋯I and H⋯O contacts, while C⋯I and N⋯I inter­actions indicate the presence of I⋯π contacts. The compound reported here represents the first example of a chiral *A*_2_Cu*X*_3_-type metal halide, which shows potential for second-harmonic generation, polarized blue-light emission, and other non-linear optical applications.

## Abstract 2

The newly synthesized compound (l-HisH_2_)CuI_3_·H_2_O (where l-HisH_2_ = l-histidinium) possesses a rare one-dimensional *A*_2_Cu*X*_3_-type structure built from chiral left-handed helical chains of corner-sharing [CuI_4_] tetra­hedra. Crystal structure and Hirshfeld surface analyses reveal that hydrogen bonding and π⋯I inter­actions play a vital role in mediating the inter­actions between the organic and inorganic components of this compound. Distortion analysis using τ_4_, τ_4_’, and Baur indices shows that this compound exhibits the lowest [CuI_4_] tetra­hedral distortion among reported analogues.

## 1. Chemical context

Recently, copper(I) halide materials have attracted significant inter­est because of their promising properties for applications in optoelectronics and radiation scintillators (Popy *et al.*, 2024 ▸; Kirakci *et al.*, 2017 ▸; Banerjee & Saparov, 2023 ▸; Chen *et al.*, 2025 ▸; Du *et al.*, 2023 ▸; Zhang *et al.*, 2024 ▸). Copper(I) halide-based materials exhibit high photoluminescence quantum yields, tunable crystal structures, and, thanks to their structural and chemical diversity, feature adjustable band gaps and photophysical properties (Banerjee & Saparov, 2023 ▸). Their tunable photoluminescence wavelengths are especially important for next-generation lighting devices (Banerjee & Saparov, 2023 ▸). Compared to the extensively studied lead-halide materials, copper-based halides also have the advantage of lower toxicity.

Copper(I) halides reported so far, with both inorganic and organic counter-ions, typically form zero-dimensional or one-dimensional structures, such as Rb_2_CuBr_3_ (Creason *et al.*, 2020 ▸), (Bmpip)_2_Cu_2_Br_4_ (where Bmpip = 1-butyl-1-methyl­piperidinium; Xu *et al.*, 2022 ▸), and PPh_4_CuBr_2_ (Xu *et al.*, 2022 ▸) and 1D-(Npipz)_2_Cu_2_I_6_ (where Npipz = 1-butyl-1-methyl­piperidinium; Carignan *et al.*, 2024 ▸). These compounds have become known as highly efficient blue-light emitters, up-conversion materials, and scintillators with large light yields, mainly because of efficient recombination pathways involving self-trapped excitons (STE).

Recently, one-dimensional copper(I) halides with the general formula *A*_2_Cu*X*_3_ have become more studied. These materials, made up of [Cu*X*_3_]_*n*_^2*n*−^ chains formed through corner-sharing [Cu*X*_4_] tetra­hedra, exhibit decent photoluminescence quantum yields and often excellent scintillation performance. Although such materials are still relatively rare, these one-dimensional copper(I) halides show halide-tunable luminescence in the lower-wavelength visible range, emitting blue, purple, and cyan light - an attractive feature for lighting and display applications (Carignan *et al.*, 2024 ▸; Du *et al.*, 2023 ▸; Zhang *et al.*, 2024 ▸).

Introducing chirality into these systems could enhance their functionality, enabling polarized light emission in the visible range and expanding their potential for nonlinear optical applications. Chiral α-amino acids, particularly l-histidine, have been shown to act as effective structure-directing agents in the synthesis of chiral metal halides, including hybrid perovskite materials (Sirenko *et al.*, 2024 ▸, 2023 ▸). l-Histidine is particularly notable because it can adopt two protonation states, existing as either the mono or diprotonated l-histidinium.
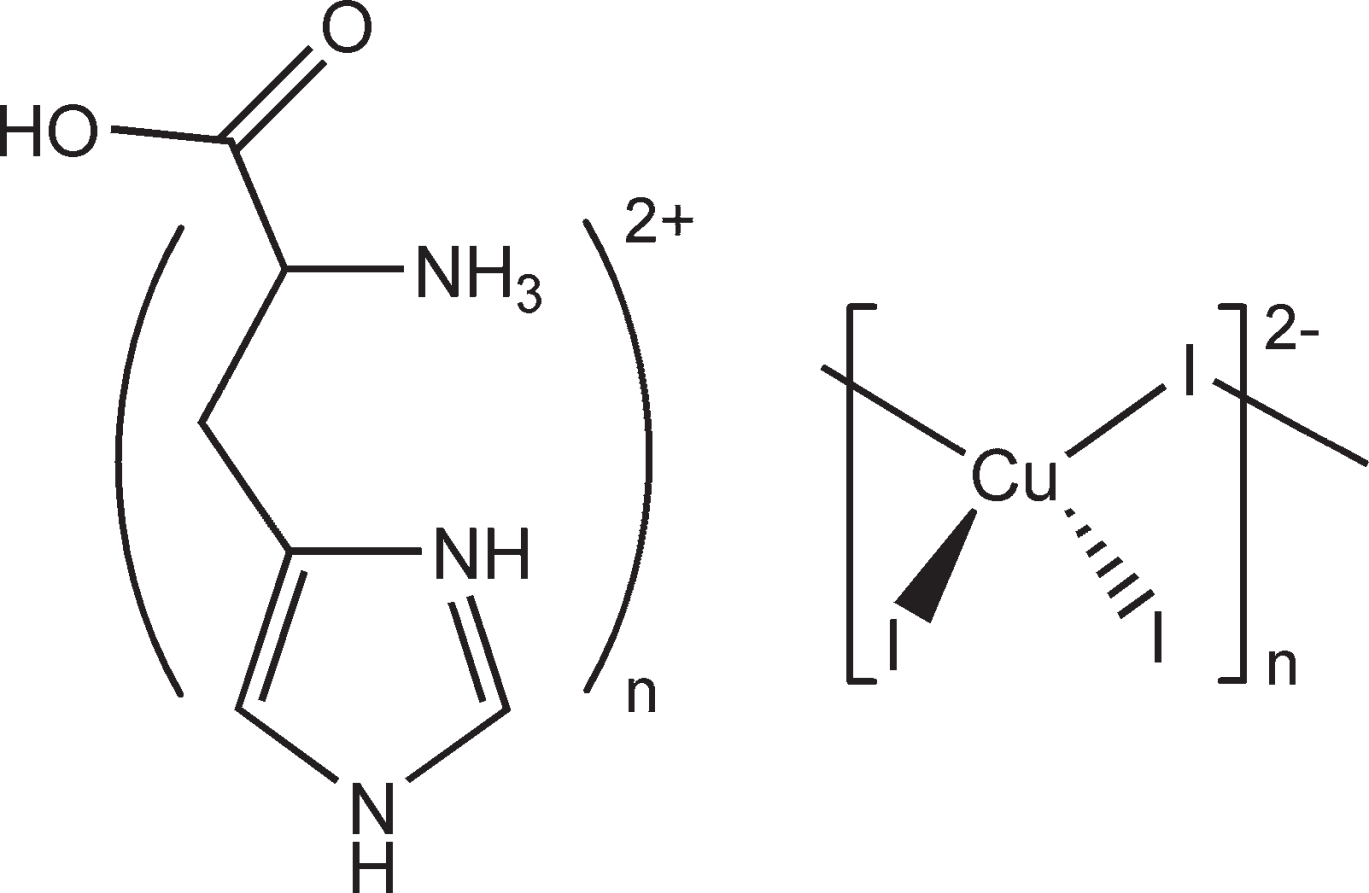


In this paper we report a new chiral low-dimensional copper(I) iodide hybrid material obtained using the reaction between l-histidine and copper(I) iodide in concentrated hydro­iodic acid. A detailed structural characterization and a Hirshfeld surface analysis were carried out for the resulting compound, (l-HisH_2_)CuI_3_·H_2_O (**1**).

## 2. Structural commentary

**Figure 1:**
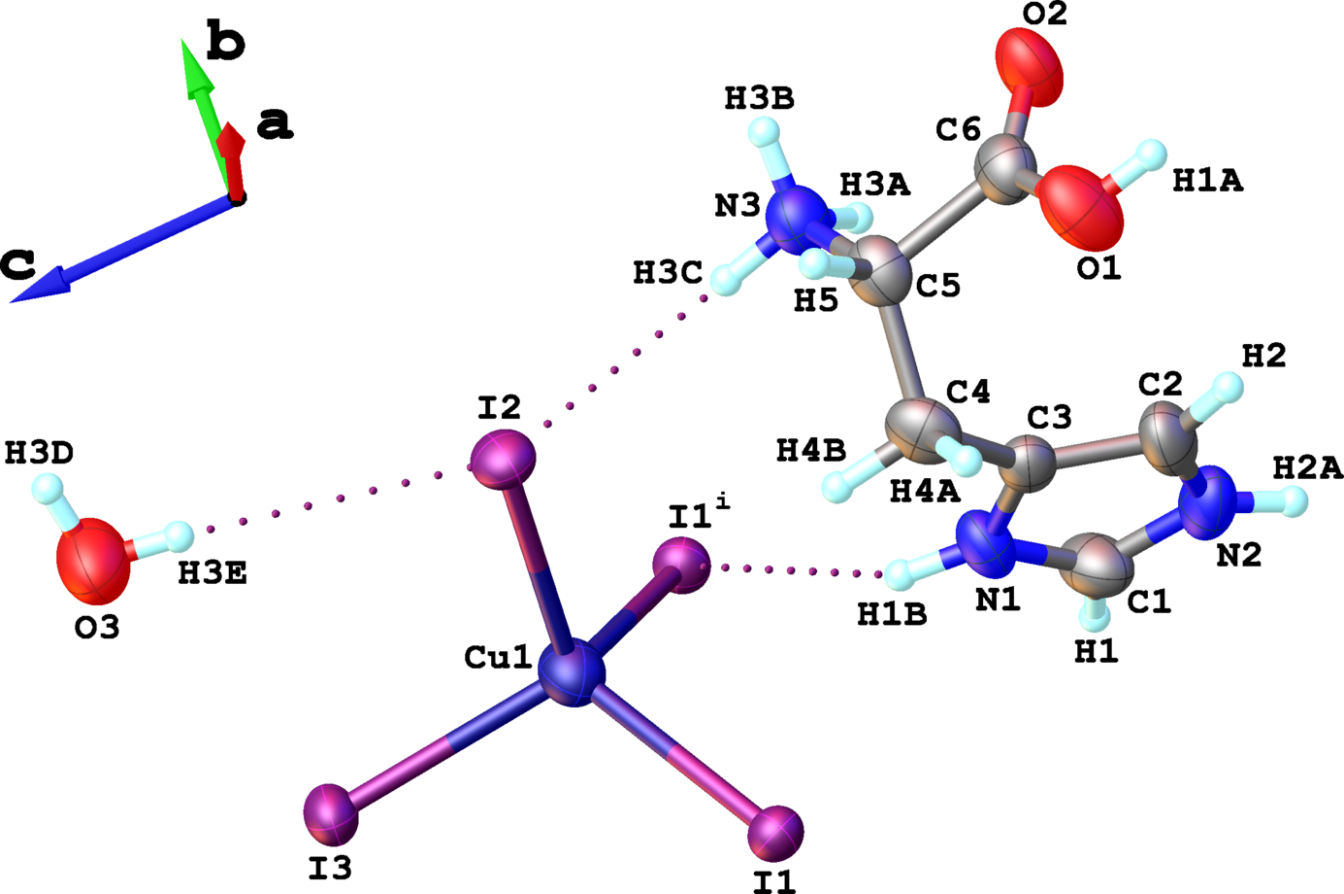
Representation of the building units in the crystal structure of **1**, showing the atom-labeling scheme. Displacement ellipsoids are drawn at the 50% probability level. H atoms are shown as small spheres of arbitrary radius. [Symmetry codes: (i) −*x*, 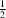 + *y*, 1 − *z*].

The title compound crystallizes in the monoclinic space group *P*2_1_. The asymmetric unit of **1** contains one l-histidinium cation, one Cu^+^ cation, three iodide anions and a co-crystallized water mol­ecule (Fig. 1 ▸). Each Cu^+^ cation coordinated by four iodide ligands adopts a tetra­hedral coordination geometry (Fig. 1 ▸). In each [CuI_4_] tetra­hedron, two iodide atoms bridge neighboring Cu^I^ centers, while the other two are terminal, inter­acting only with Cu^I^ and forming hydrogen bonds with the l-histidinium cations and co-crystallized water mol­ecules (Fig. 1 ▸). The Cu—I bond lengths in the [CuI_4_] coordination tetra­hedra range from 2.62 to 2.72 Å (Table 1 ▸), which is similar to values observed in other *A*_2_CuI_3_-type compounds reported to date (Zhang *et al.*, 2024 ▸; Carignan *et al.*, 2024 ▸; Du *et al.*, 2023 ▸). The I—Cu—I bond angles in the [CuI_4_] tetra­hedra range from 106.19 to 113.21°, deviating from the ideal tetra­hedral value of ∼109.5° and demonstrating a smaller angle deviation compared to the previously reported compounds (Zhang *et al.*, 2024 ▸; Carignan *et al.*, 2024 ▸; Du *et al.*, 2023 ▸). In **1**, the Cu—I bonds with the terminal iodides (∼2.63 Å) are shorter than those with the bridging μ-iodides (∼2.71 Å), consistent with previously reported *A*_2_CuI_3_ compounds (Zhang *et al.*, 2024 ▸; Carignan *et al.*, 2024 ▸; Du *et al.*, 2023 ▸). The Cu—μ-I—Cu bridging angle between adjacent tetra­hedra is 125.79 (3)°, notably larger than the ∼108° observed in other A_2_CuI_3_ compounds (Zhang *et al.*, 2024 ▸; Carignan *et al.*, 2024 ▸; Du *et al.*, 2023 ▸).

A convenient way to describe distortions in coordination polyhedra is by using distortion indices. Several indices have been introduced in the literature, particularly for metal–oxygen tetra­hedra. Baur proposed the following distortion indices for metal–oxide tetra­hedra (Baur, 1970 ▸):

DI(*AX*) = Σ^4^_*i*=1_ | (*A* – *X*)_*i*_ – <*A* – *X*> | / (4 < *A* – *X* >),

DI(*XAX*) = Σ^6^_*i*=1_ | (*X* – *A* – *X*)_*i*_ – < *X* – *A* – *X* > | / (6 < *X* – *A* – *X* >)

and

DI(*XX*) = Σ^6^_*i*=1_ | (*X*⋯*X*)_*i*_ – < *X*⋯*X* > | / (6 < *X*⋯*X* >).

Here, DI(*AX*), DI(*XX*) and DI(*XAX*) represent the bond-length distortion parameter (BLDP), edge-length distortion parameter (ELDP) and bond-angle distortion parameter (BADP), respectively, which together provide a complete description of the distortion of coordination tetra­hedra. Although these indices were originally developed for ionic metal–oxygen systems, they can also be applied to tetra­hedra with Cu—I bonds, which have partial covalent character. The calculated values for compound **1** are DI(*AX*) = 0.0152, DI(*XAX*) = 0.01608, and DI(*XX*) = 0.01615 (Table 2 ▸). Among *A*_2_CuI_3_-type compounds, **1** shows the highest DI(*AX*) value, whereas its DI(*XAX*) and DI(*XX*) values are the lowest reported for this family. Additionally, the parameter τ_4_ which was developed for four-coordinated structures, is often used to describe deviations from ideal tetra­hedral geometry (Yang *et al.*, 2007 ▸). For square planar structures, τ_4_ = 0, whereas for tetra­hedral structures, τ_4_ = 1:

τ_4_ = [360° – (α + β)] / [360° – 2θ],

where β is the largest and α is the second-largest bond angle (β > α) in a four-coordinate geometry, and θ = arccos(–1/3) ≈ 109.5° is the ideal tetra­hedral angle.

This parameter was later refined to more effectively distinguish between four-coordinate complexes that have significantly different geometries but similar τ_4_ values. The revised parameter, τ_4_′ (Okuniewski *et al.*, 2015 ▸), provides values comparable to τ_4_ but offers improved discrimination among the examined structures (τ_4_′ > τ_4_):

τ_4_′ = [(β – α) / (360° – θ)] + [(180° – β) / (180° – θ)]

(α, β and θ as before). For **1**, τ_4_ and τ_4_′ are 0.965 and 0.957, respectively, indicating a slight distortion of the [CuI_4_] tetra­hedra relative to the ideal geometry (τ_4_ = τ_4_′ = 1) (Table 2 ▸). These values are the highest reported among related one-dimensional copper(I) halides, suggesting that the [CuI_4_] tetra­hedra in **1** are the least distorted within this family (Table 2 ▸).

**Figure 2:**
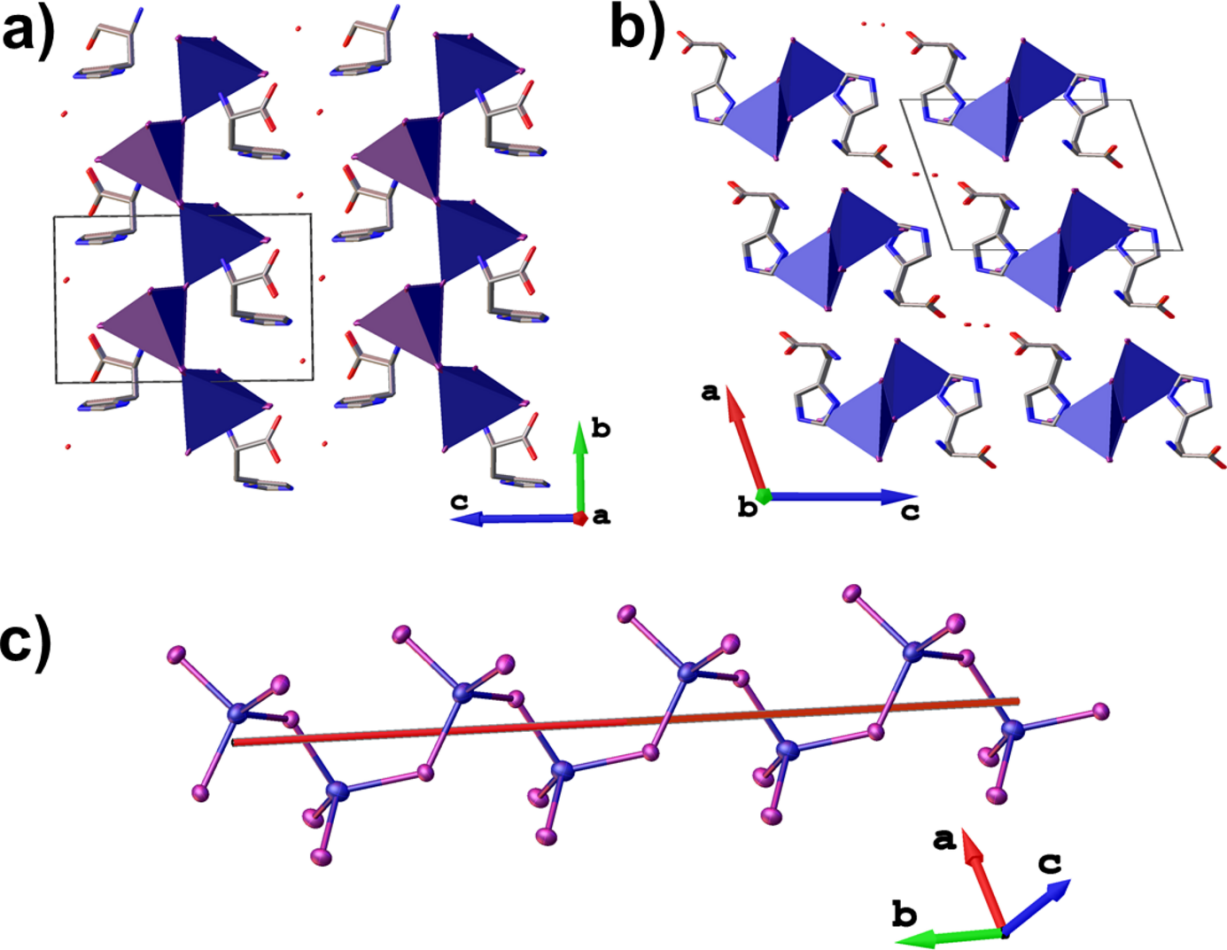
Crystal packing of **1** viewed along (*a*) the [100] and (*b*) the [010] directions, and (*c*) side view of a fragment of the crystal structure showing an inorganic chain with left-handed helical chirality.

The [CuI_4_] coordination polyhedra participate in both μ_2_-bridging coordination (Cu—μ-I—Cu) and hydrogen bonding with the l-histidinium cations, forming infinite [CuI_3_]_*n*_^2*n*−^ polymeric chains that propagate along the [010] direction (Fig. 2 ▸). Inter­estingly, the [CuI_3_]_*n*_^2*n*−^ chains (point group 2) exhibit left-handed helical chirality, suggesting a structure-directing role of the chiral l-histidinium cations in the formation of these helical chains (Fig. 2 ▸*c*). Moreover, the Cu⋯Cu distance of 4.83 Å between adjacent tetra­hedra is the largest reported among compounds featuring one-dimensional chains of corner-sharing [CuI_4_] tetra­hedra (Zhang *et al.*, 2024 ▸; Carignan *et al.*, 2024 ▸; Du *et al.*, 2023 ▸).

## 3. Supra­molecular features

**Figure 3:**
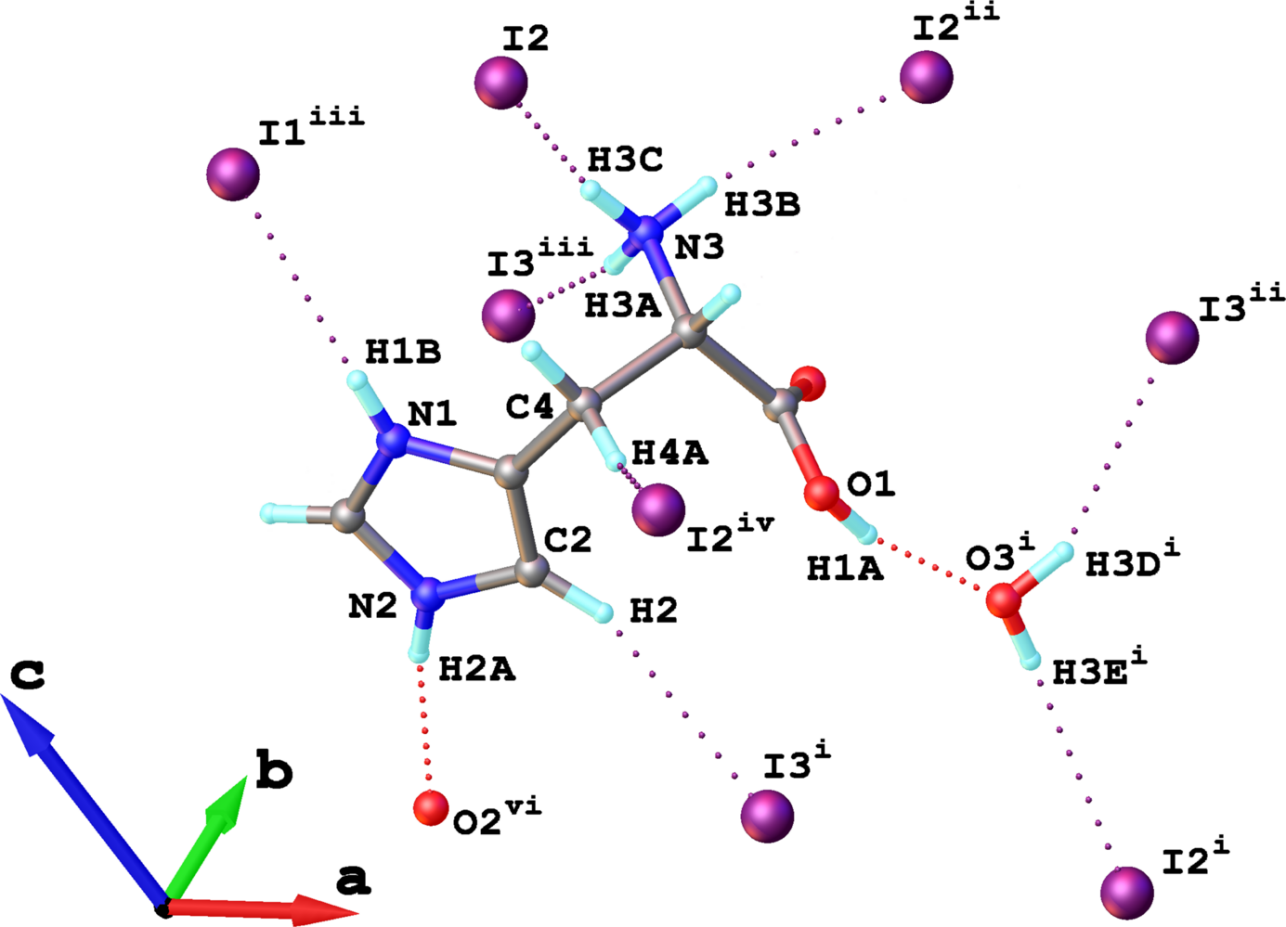
Side views of a fragment of the crystal structure of **1**, illustrating the hydrogen-bonding scheme of the l-histidinium cation (dotted lines). [Symmetry codes: (i) *x*, *y*, −*z* + 1; (ii) −*x* + 1, *y* + 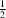, −*z* + 1; (iii) −*x*, *y* + 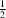, −*z* + 1; (iv) −*x* + 1, *y* − 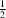, −*z* + 1; (v) −*x* + 1, *y*, *z*; (vi) −*x*, 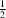 + *y*, −*z*].

The l-histidinium cations and co-crystallized water mol­ecules inter­act with the one-dimensional helical chains of corner-sharing [CuI_4_] tetra­hedra through a network of N—H⋯I and O—H⋯I hydrogen bonds, along with weak C—H⋯I contacts (Fig. 3 ▸, Table 3 ▸). In **1**, the l-histidinium cation is doubly protonated at both the imidazolium ring and the amino group, which enables the formation of the *A*_2_Cu*X*_3_-type structure. The protonated amino group participates in N—H⋯I hydrogen bonding with two adjacent [CuI_3_]_*n*_^2−^ chains. Specifically, two hydrogen bonds, N3_(a)_—H3*B*_(a)_⋯I2^ii^ and N3_(a)_—H3*C*_(a)_⋯I2 [subscript (a) denotes the amino group], connect the l-histidinium cation to one chain, while N3_(a)_—H3*A*_(a)_⋯I3^iii^ links it to a second chain (Fig. 3 ▸). The protonated imidazolium ring contributes to linking neighboring [CuI_3_]_*n*_^2*n*−^ chains *via* N1_(i)_—H1*B*_(i)_⋯I1^iii^ hydrogen bonds [where (i) denotes the imidazolium ring] (Table 3 ▸, Fig. 3 ▸). The carboxyl group of the L-histidinium cation and the co-crystallized water mol­ecules further consolidate the organic—inorganic framework through hydrogen-bonding inter­actions. In particular, the hydroxyl group of the carboxyl participates in O1_(c)_—H1*A*_(c)_⋯O3_(w)_^i^ hydrogen bonding with the co-crystallized water mol­ecules (Fig. 3 ▸), where (w) denotes co-crystallized water and (c) denotes the carboxyl group. The co-crystallized water mol­ecule participates in O3_(w)_^i^—H3*E*^i^⋯I2^i^ and O3_(w)_^i^—H3*D*^i^⋯I3^ii^ hydrogen bonding with two inorganic chains (Fig. 3 ▸, Table 3 ▸). The l-histidinium cations inter­act with each other through an N2_(i)_—H2*A*_(i)_⋯O2_(c)_^vi^ hydrogen bond (Fig. 3 ▸), which links the imidazolium N—H group of one cation to the carbonyl oxygen of the carboxyl group of another cation.

**Figure 4:**
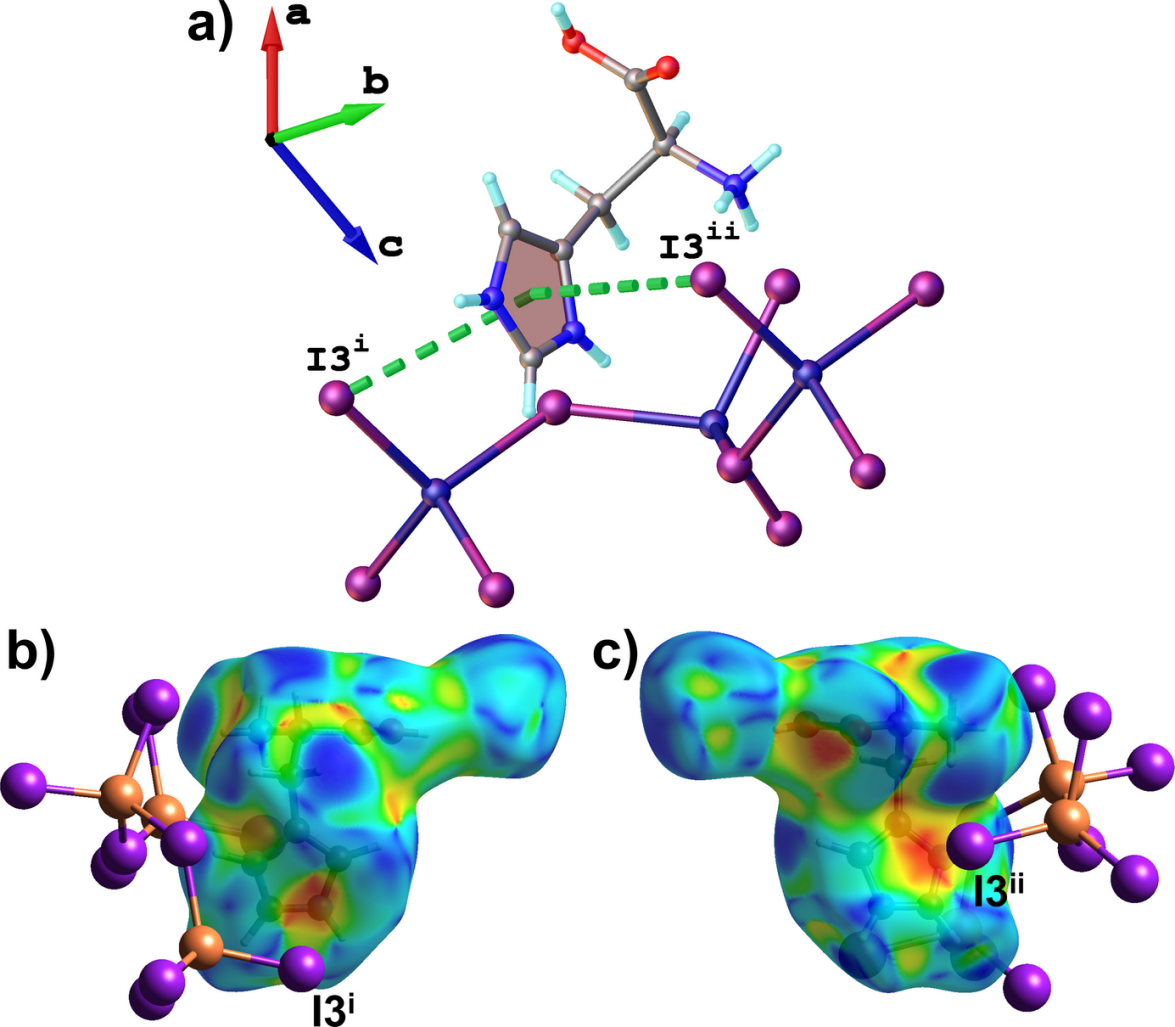
(*a*) Side views of a fragment of the crystal structure of **1**, illustrating I⋯π inter­actions (green dashed line). (*b*), (*c*) The Hirshfeld surface mapped with the shape-index function highlights I⋯π inter­actions between I3^i^ and I3^ii^ atoms and the imidazolium ring. [Symmetry codes: (i) −*x*, *y* − 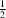, 1 − *z*; (ii) −*x*, *y* + 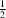, −*z* + 1].

Furthermore, the secondary CH_2_ group of the aliphatic backbone of l-histidinium also participate in weak C4—H4*A*⋯I2^iv^ (Fig. 3 ▸) contacts, further reinforcing the cohesion between the organic and inorganic components of **1**. As previously established, C—H⋯*B* (where *B* denotes the hydrogen bond acceptor) hydrogen bonding can be considered when (*r* C⋯B) – [*r*_vdW_(*B*) + *r* C—H] < 1.00 Å, where *r* C—H is the average C—H bond length, and *r*_vdW_(*B*) is the van der Waals radius of the hydrogen-bond acceptor (Harmon *et al.*, 1992 ▸). For the weak C2—H2⋯I3^i^ contacts (Fig. 3 ▸) involving the imidazolium ring, the (*r* C⋯*B*) – [*r*_vdW_(*B*) + *r* C—H] difference is 0.91 Å with a bond angle of 157°, which lies within the expected range for such inter­actions. For C4—H4*A*⋯I2^iv^ weak contact (Fig. 3 ▸) (secondary CH_2_ group), the difference is 0.686 Å with a bond angle of 145°, indicating weak hydrogen bonding. Moreover, the imidazolium moiety of l-histidinium participates in an I⋯π inter­action (Fig. 4 ▸) with the I3 atom of [CuI_4_] unit, with centroid⋯I3^i^ and centroid⋯I3^ii^ distances of of 4.056 (4) and 3.697 (4) Å, respectively, and an I3^i^⋯centroid⋯I3^ii^ angle of 155.36 (12)°, which falls within the range previously reported for I⋯π inter­actions (Prasanna & Guru Row, 2000 ▸). Concave red regions on the Hirshfeld surface mapped with the shape-index function (Fig. 4 ▸*b*,*c*) further indicate the presence of I⋯π inter­actions in the compound.

## 4. Hirshfeld surface analysis

**Figure 5:**
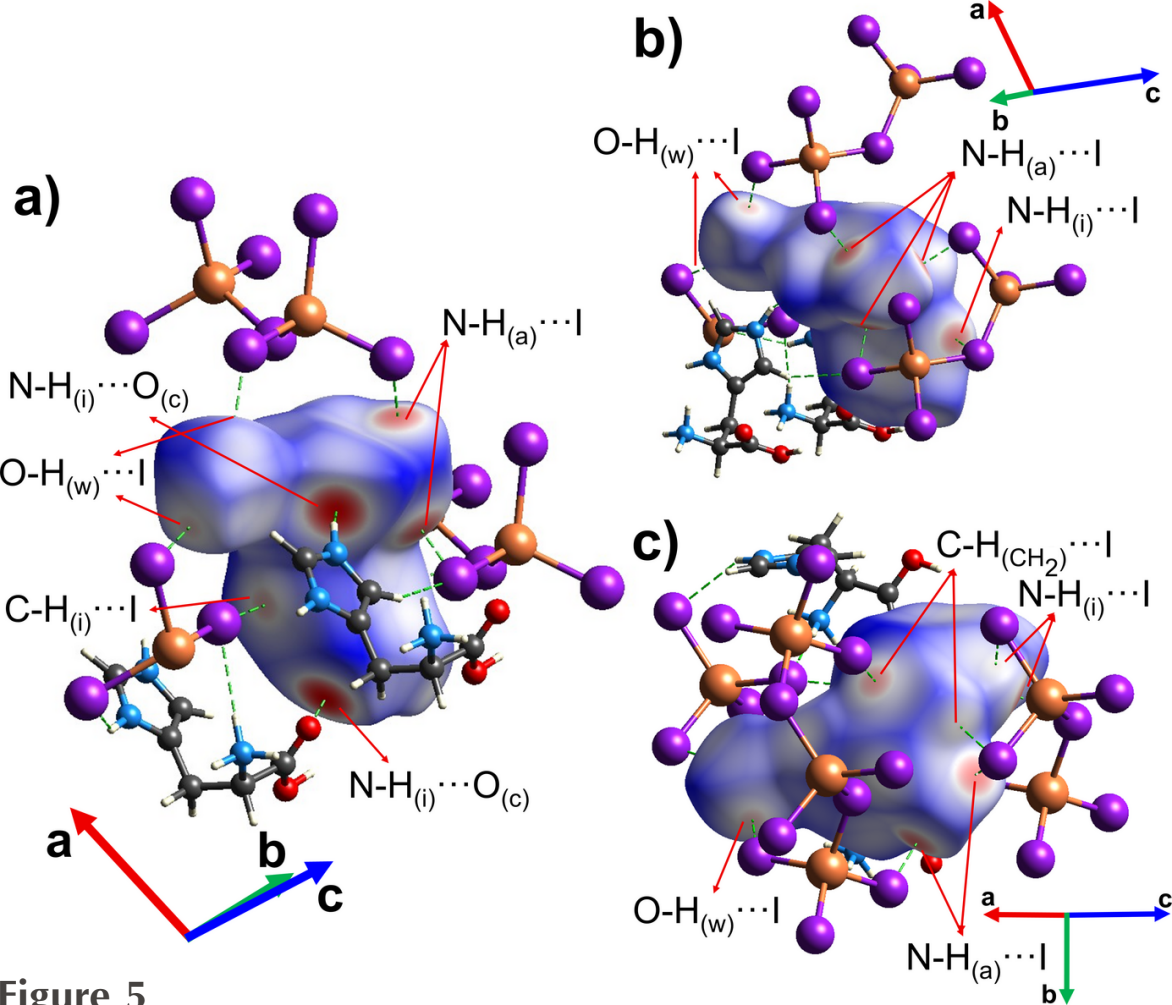
(*a*)–(*c*) Representation of the Hirshfeld surface of the l-histidinium cation in **1** along different crystallographic directions, with the *d*_norm_ function plotted onto the surface to highlight various inter­actions. The subscripts indicate different functional groups: (a) = NH_3_^+^; (c) = COOH; (i) = imidazolium ring; (w) = co-crystallized water mol­ecule; (CH_2_) = methyl­ene group.

**Figure 6:**
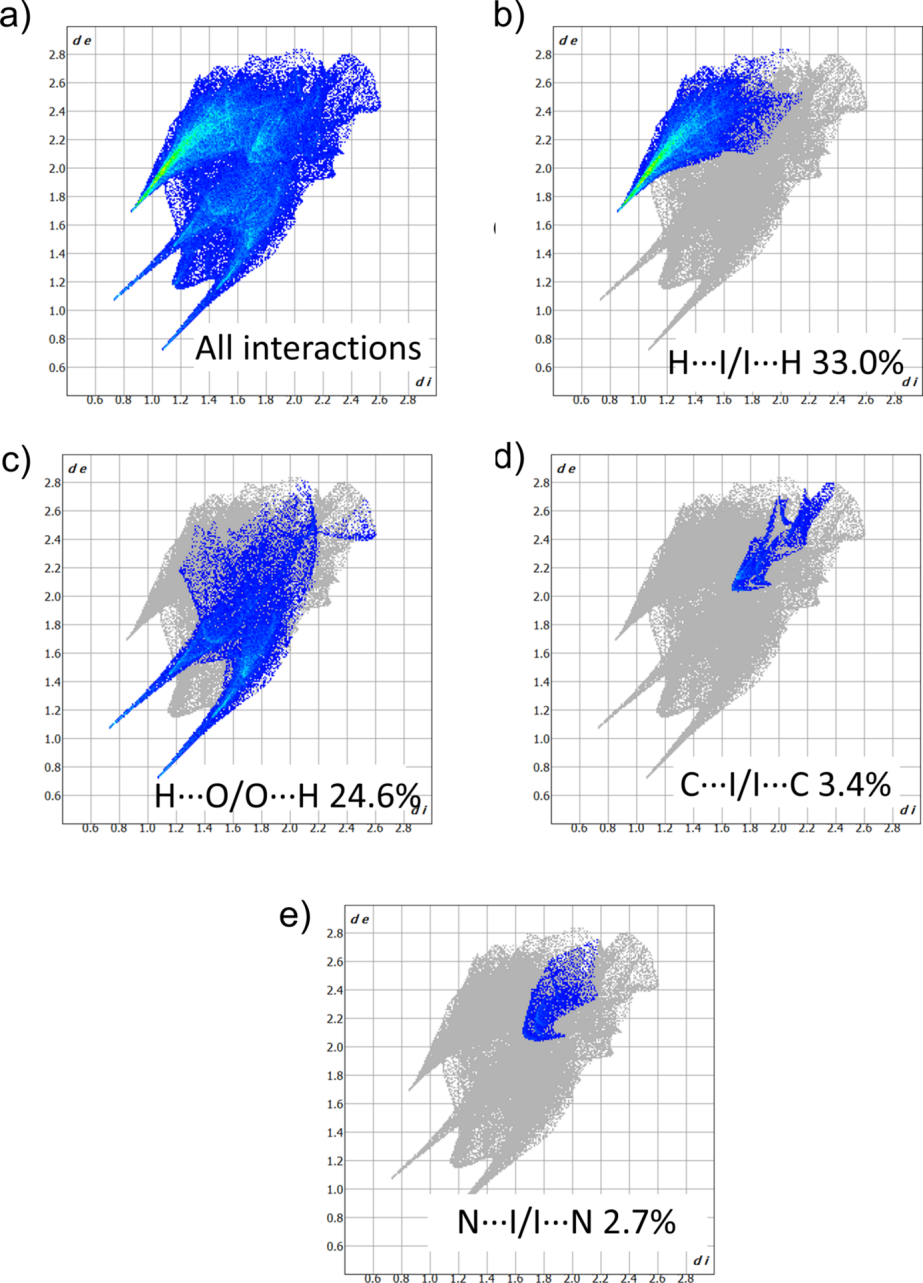
Two-dimensional fingerprint plots from a Hirshfeld surface analysis of **1** showing: (*a*) all contacts; (*b*) H⋯I/I⋯H (33.0%); (*c*) H⋯O/O⋯H (24.6%); (*d*) C⋯I/I⋯C (3.4%); (*e*) N⋯I/I⋯N (2.7%).

The Hirshfeld surface and the corresponding two-dimensional fingerprint plots were generated for the fragment containing the l-histidinium cation and the co-crystallized water mol­ecule using *CrystalExplorer 21.5* (Spackman *et al.*, 2021 ▸) with standard resolution for the three-dimensional *d*_norm_ surfaces (Figs. 5 ▸ and 6 ▸). The red spots on the Hirshfeld surface are attributed to hydrogen bonds and weak C—H⋯I contacts between this fragment and both the [CuI_3_]_*n*_^2*n*−^ anionic chains and other l-histidinium cations (only hydrogen bonds) (Fig. 5 ▸). The associated fingerprint plots (Fig. 6 ▸) confirm that hydrogen bonding dominates the crystal packing of **1**. The analysis shows that H⋯I inter­actions are predominant (Fig. 6 ▸*b*), accounting for approximately 33% of the total Hirshfeld surface. These correspond to N—H⋯I and O—H⋯I hydrogen bonds, as well as the weaker C—H⋯I contacts that link the organic and inorganic components. H⋯O contacts are the second most significant, contributing approximately 25% (Fig. 6 ▸*c*) to the Hirshfeld surface, and arise from O—H⋯O and N—H⋯O hydrogen bonds. The contribution of C⋯I and N⋯I contacts (less than 5%) to the Hirshfeld surface indicates the presence of I⋯π inter­actions (Fig. 6 ▸*d* and 6*e*), which further consolidate the crystal packing of **1**.

## 5. Database survey

A search of the Cambridge Structure Database (CSD version 6.00, last update August 2025; Groom *et al.*, 2016 ▸) revealed 614 structures for the [CuI_4_] moiety. Most similar to the title compound, namely complexes containing one-dimensional [CuI_3_]_*n*_^2*n*−^ chains of corner-shared tetra­hedra, are *catena*-[1-methyl­piperazine-1,4-diium (μ-iodo)­diiodo­copper] (BOK­LEW; Carignan *et al.*, 2024 ▸), *catena*-[propane-1,2-di­ammonium (μ-iodo)­bis­(iodo)­dicopper(I)] (FOSMAF; Zhang *et al.*, 2024 ▸), and *catena*-[propane-1,3-bis­(ammonium) (μ-iodo)-bis­(iodo)­copper(I)] (MISJAD; Du *et al.*, 2023 ▸).

## 6. Synthesis and crystallization

l-histidine (20 mg, 0.129 mmol) and copper(I) iodide (25 mg, 0.131 mmol) were dissolved in 600 µL of concentrated HI (57%) and 25 µL of H_3_PO_2_. The resulting mixture was left to evaporate, and after 2 weeks, colorless, plate-like crystals of the (L-HisH_2_)CuI_3_·H_2_O compound were obtained. These crystals were placed under crystallographic oil until further single crystal X-ray diffraction measurements.

## 7. Refinement details

Crystal data, data collection, and structure refinement details are summarized in Table 4 ▸. All H atoms were placed geometrically and refined as riding, with C—H = 0.98 Å and *U*_iso_(H) = 1.2*U*_eq_(C) for ternary CH; C—H = 0.97 Å and *U*_iso_(H) = 1.2*U*_eq_(C) for secondary CH_2_; N—H = 0.86 Å and *U*_iso_(H) = 1.2*U*_eq_(N) for aromatic NH; C—H = 0.93 Å and *U*_iso_(H) = 1.5*U*_eq_(C) for aromatic CH groups; O—H = 0.86 Å and *U*_iso_(H) = 1.5*U*_eq_(O) for water mol­ecules. Amino H atoms were positioned geometrically and allowed to ride on N atoms and rotate around the C—N bond, with N—H = 0.89 Å and *U*_iso_(H) = 1.5*U*_eq_(N) for NH_3_ groups. Carboxyl­ate H atoms were positioned geometrically and allowed to ride on O atoms and rotate around the O—C bond, O_(c)_—H = 0.82 Å (c = carboxyl­ate) and *U*_iso_(H) = 1.5*U*_eq_(O) for the O_(c)_—H groups of carboxyl­ate.

## Supplementary Material

Crystal structure: contains datablock(s) I. DOI: 10.1107/S2056989025010023/oi2028sup1.cif

Structure factors: contains datablock(s) I. DOI: 10.1107/S2056989025010023/oi2028Isup2.hkl

CCDC reference: 2501923

Additional supporting information:  crystallographic information; 3D view; checkCIF report

Additional supporting information:  crystallographic information; 3D view; checkCIF report

## Figures and Tables

**Table 1:** Selected geometric parameters (Å, °)

I1—Cu1^i^	2.7063 (13)	I2—Cu1	2.6225 (12)
I1—Cu1	2.7216 (13)	I3—Cu1	2.6433 (11)
			
Cu1^i^—I1—Cu1	125.80 (3)	I2—Cu1—I3	106.19 (4)
I1^ii^—Cu1—I1	109.20 (4)	I3—Cu1—I1^ii^	110.75 (4)
I2—Cu1—I1	107.70 (4)	I3—Cu1—I1	113.21 (5)
I2—Cu1—I1^ii^	109.68 (5)		

Symmetry codes: (i) 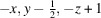; (ii) 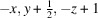.

**Table 2:** Selected octa­hedral distortion parameters

	L-HisH_2_[CuI_3_]·H_2_O^*a*^	1D-(Npipz)_2_Cu_2_I_6_^*b*^	[1,2-PDA]CuI_3_^*c*^	[1,3-PDA]CuI_3_^*d*^
DI(*AX*)	0.0152	0.00941	0.00927	0.02204
DI(*XAX*)	0.01608	0.04301	0.02587	0.03077
DI(*XX*)	0.01615	0.03742	0.01557	0.01237
τ_4_	0.965	0.937	0.941	0.931
τ_4_′	0.957	0.934	0.935	0.914

(*a*) The title compound (**1**); (*b*) Carignan *et al.* (2024 ▸); (*c*) Du *et al.* (2023 ▸); (*d*) Zhang *et al.* (2024 ▸).

**Table 3:** Hydrogen-bond geometry (Å, °)

*D*—H⋯*A*	*D*—H	H⋯*A*	*D*⋯*A*	*D*—H⋯*A*
O3—H3*D*⋯I3^iii^	0.86	3.02	3.768 (8)	146
O3—H3*E*⋯I2	0.82	3.12	3.938 (7)	177
O1—H1*A*⋯O3^iv^	0.82	1.81	2.605 (10)	162
N3—H3*A*⋯I3^ii^	0.89	2.69	3.571 (8)	169
N3—H3*B*⋯I2^v^	0.89	2.75	3.601 (8)	160
N3—H3*C*⋯I2	0.89	2.79	3.596 (7)	152
N1—H1*B*⋯I1^ii^	0.86	2.81	3.516 (7)	141
N2—H2*A*⋯O2^vi^	0.86	1.97	2.804 (9)	163
C4—H4*A*⋯I2^vii^	0.97	2.88	3.716 (9)	146

Symmetry codes: (ii) 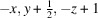; (iii) 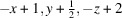; (iv) 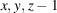; (v) 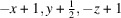; (vi) 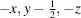; (vii) 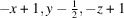.

**Table 4:** Experimental details

Crystal data	
Chemical formula	(C_6_H_11_N_3_O_2_)[CuI_3_]·H_2_O
*M*_r_	619.43
Crystal system, space group	Monoclinic, *P*2_1_
Temperature (K)	299
*a*, *b*, *c* (Å)	8.5704 (3), 7.5748 (2), 12.3860 (4)
β (°)	109.113 (3)
*V* (Å^3^)	759.76 (4)
*Z*	2
Radiation type	Mo *K*α
μ (mm^−1^)	7.53
Crystal size (mm)	0.18 × 0.15 × 0.12

Data collection	
Diffractometer	XtaLAB Synergy, Dualflex, HyPix
Absorption correction	Analytical (*CrysAlis PRO*; Rigaku OD, 2024 ▸)
*T*_min_, *T*_max_	0.398, 0.517
No. of measured, independent and observed [*I* > 2σ(*I*)] reflections	10397, 3728, 3615
*R*_int_	0.025
(sin θ/λ)_max_ (Å^−1^)	0.710

Refinement	
*R*[*F*^2^ > 2σ(*F*^2^)], *wR*(*F*^2^), *S*	0.029, 0.073, 1.05
No. of reflections	3728
No. of parameters	147
No. of restraints	1
H-atom treatment	H-atom parameters constrained
Δρ_max_, Δρ_min_ (e Å^−3^)	0.98, −0.86
Absolute structure	Flack *x* determined using 1510 quotients [(*I*^+^)-(*I*^-^)]/[(*I*^+^)+(*I*^-^)] (Parsons *et al.*, 2013 ▸)
Absolute structure parameter	−0.03 (4)

Computer programs: *CrysAlis PRO* (Rigaku OD, 2024 ▸), *SHELXT2018/2* (Sheldrick, 2015*a* ▸), *SHELXL2019/3* (Sheldrick, 2015*b* ▸) and *OLEX2* (Dolomanov *et al.*, 2009 ▸).

## supplementary crystallographic information

### catena-Poly[4-[(2S)-2-azaniumyl-2-carboxyethyl]-1H-imidazol-3-ium [[diiodidocuprate(I)]-µ-iodido] monohydrate] Crystal data

**Table:**

(C_6_H_11_N_3_O_2_)[CuI_3_]·H_2_O	*F*(000) = 564
*M**_r_* = 619.43	*D*_x_ = 2.708 Mg m^−^^3^
Monoclinic, *P*2_1_	Mo *K*α radiation, λ = 0.71073 Å
*a* = 8.5704 (3) Å	Cell parameters from 8127 reflections
*b* = 7.5748 (2) Å	θ = 2.6–30.0°
*c* = 12.3860 (4) Å	µ = 7.53 mm^−^^1^
β = 109.113 (3)°	*T* = 299 K
*V* = 759.76 (4) Å^3^	Plate, clear intense colourless
*Z* = 2	0.18 × 0.15 × 0.12 mm

### catena-Poly[4-[(2S)-2-azaniumyl-2-carboxyethyl]-1H-imidazol-3-ium [[diiodidocuprate(I)]-µ-iodido] monohydrate] Data collection

**Table:**

XtaLAB Synergy, Dualflex, HyPix diffractometer	3728 independent reflections
Radiation source: micro-focus sealed X-ray tube, PhotonJet (Mo) X-ray Source	3615 reflections with *I* > 2σ(*I*)
Mirror monochromator	*R*_int_ = 0.025
Detector resolution: 10.0000 pixels mm^-1^	θ_max_ = 30.3°, θ_min_ = 2.5°
ω scans	*h* = −11→11
Absorption correction: analytical (CrysAlisPro; Rigaku OD, 2024)	*k* = −10→10
*T*_min_ = 0.398, *T*_max_ = 0.517	*l* = −15→17
10397 measured reflections	

### catena-Poly[4-[(2S)-2-azaniumyl-2-carboxyethyl]-1H-imidazol-3-ium [[diiodidocuprate(I)]-µ-iodido] monohydrate] Refinement

**Table:**

Refinement on *F*^2^	Hydrogen site location: mixed
Least-squares matrix: full	H-atom parameters constrained
*R*[*F*^2^ > 2σ(*F*^2^)] = 0.029	*w* = 1/[σ^2^(*F*_o_^2^) + (0.0449*P*)^2^ + 0.2675*P*] where *P* = (*F*_o_^2^ + 2*F*_c_^2^)/3
*wR*(*F*^2^) = 0.073	(Δ/σ)_max_ < 0.001
*S* = 1.05	Δρ_max_ = 0.98 e Å^−^^3^
3728 reflections	Δρ_min_ = −0.86 e Å^−^^3^
147 parameters	Absolute structure: Flack *x* determined using 1510 quotients [(*I*^+^)-(*I*^-^)]/[(*I*^+^)+(*I*^-^)] (Parsons *et al.*, 2013)
1 restraint	Absolute structure parameter: −0.03 (4)
Primary atom site location: dual	

### catena-Poly[4-[(2S)-2-azaniumyl-2-carboxyethyl]-1H-imidazol-3-ium [[diiodidocuprate(I)]-µ-iodido] monohydrate] Special details

**Table:**

Geometry. All esds (except the esd in the dihedral angle between two l.s. planes) are estimated using the full covariance matrix. The cell esds are taken into account individually in the estimation of esds in distances, angles and torsion angles; correlations between esds in cell parameters are only used when they are defined by crystal symmetry. An approximate (isotropic) treatment of cell esds is used for estimating esds involving l.s. planes.

### catena-Poly[4-[(2S)-2-azaniumyl-2-carboxyethyl]-1H-imidazol-3-ium [[diiodidocuprate(I)]-µ-iodido] monohydrate] Fractional atomic coordinates and isotropic or equivalent isotropic displacement parameters (Å^2^)

**Table:**

	*x*	*y*	*z*	*U*_iso_*/*U*_eq_	
I1	0.13680 (5)	0.06749 (6)	0.50811 (4)	0.03160 (12)	
I2	0.40061 (6)	0.55289 (7)	0.62736 (4)	0.03880 (13)	
I3	0.13407 (6)	0.34038 (8)	0.82833 (4)	0.03910 (14)	
Cu1	0.13059 (14)	0.37960 (17)	0.61550 (9)	0.0467 (3)	
C1	−0.1623 (11)	0.3476 (15)	0.1876 (8)	0.049 (2)	
H1	−0.265910	0.333504	0.195383	0.059*	
C5	0.3636 (9)	0.5864 (13)	0.3020 (6)	0.0395 (17)	
H5	0.477608	0.580101	0.354083	0.047*	
C4	0.2814 (11)	0.4064 (12)	0.3052 (8)	0.045 (2)	
H4A	0.346960	0.315430	0.285442	0.054*	
H4B	0.284618	0.384334	0.383101	0.054*	
C3	0.1048 (10)	0.3873 (10)	0.2276 (6)	0.0340 (15)	
C2	0.0371 (12)	0.3698 (14)	0.1142 (7)	0.047 (2)	
H2	0.092239	0.372943	0.060802	0.057*	
O3	0.5032 (10)	0.6129 (13)	0.9593 (6)	0.071 (2)	
H3D	0.580076	0.691853	0.978808	0.107*	
H3E	0.481288	0.605276	0.889629	0.107*	
O2	0.3119 (9)	0.7716 (9)	0.1368 (5)	0.0524 (16)	
O1	0.4420 (10)	0.5140 (11)	0.1428 (6)	0.0608 (19)	
H1A	0.439416	0.541704	0.078238	0.091*	
N3	0.2782 (10)	0.7316 (10)	0.3429 (6)	0.0433 (16)	
H3A	0.177322	0.746996	0.293110	0.065*	
H3B	0.335656	0.831167	0.349050	0.065*	
H3C	0.270935	0.702959	0.410742	0.065*	
C6	0.3693 (10)	0.6362 (13)	0.1839 (7)	0.0416 (18)	
N1	−0.0242 (9)	0.3721 (10)	0.2707 (5)	0.0410 (15)	
H1B	−0.014808	0.377986	0.341854	0.049*	
N2	−0.1306 (9)	0.3461 (11)	0.0917 (5)	0.0479 (17)	
H2A	−0.202496	0.332496	0.024939	0.057*	

### catena-Poly[4-[(2S)-2-azaniumyl-2-carboxyethyl]-1H-imidazol-3-ium [[diiodidocuprate(I)]-µ-iodido] monohydrate] Atomic displacement parameters (Å^2^)

**Table:**

	*U*^11^	*U*^22^	*U*^33^	*U*^12^	*U*^13^	*U*^23^
I1	0.0338 (2)	0.0288 (2)	0.0311 (2)	−0.00231 (19)	0.00916 (16)	−0.00163 (18)
I2	0.0332 (2)	0.0415 (3)	0.0409 (3)	−0.0090 (2)	0.01095 (18)	−0.0002 (2)
I3	0.0453 (3)	0.0436 (3)	0.0279 (2)	−0.0090 (2)	0.01119 (18)	−0.0014 (2)
Cu1	0.0450 (6)	0.0545 (6)	0.0418 (5)	−0.0078 (5)	0.0160 (4)	0.0006 (5)
C1	0.044 (4)	0.053 (5)	0.050 (5)	−0.007 (4)	0.012 (4)	0.009 (5)
C5	0.029 (3)	0.050 (5)	0.037 (4)	0.008 (3)	0.007 (3)	0.005 (4)
C4	0.042 (4)	0.044 (5)	0.048 (5)	0.013 (3)	0.013 (4)	0.014 (4)
C3	0.041 (4)	0.027 (3)	0.032 (4)	0.003 (3)	0.010 (3)	0.001 (3)
C2	0.063 (6)	0.049 (5)	0.032 (4)	0.006 (4)	0.019 (4)	−0.002 (4)
O3	0.078 (5)	0.088 (6)	0.051 (4)	−0.022 (4)	0.026 (4)	−0.006 (4)
O2	0.064 (4)	0.056 (4)	0.036 (3)	0.015 (3)	0.013 (3)	0.009 (3)
O1	0.065 (4)	0.072 (5)	0.055 (4)	0.019 (4)	0.033 (3)	0.012 (3)
N3	0.047 (4)	0.046 (4)	0.038 (4)	0.004 (3)	0.015 (3)	0.002 (3)
C6	0.036 (4)	0.049 (5)	0.036 (4)	−0.001 (3)	0.006 (3)	0.001 (3)
N1	0.049 (4)	0.046 (4)	0.029 (3)	0.008 (3)	0.014 (3)	0.006 (3)
N2	0.054 (4)	0.044 (4)	0.035 (3)	0.001 (4)	−0.001 (3)	−0.003 (3)

### catena-Poly[4-[(2S)-2-azaniumyl-2-carboxyethyl]-1H-imidazol-3-ium [[diiodidocuprate(I)]-µ-iodido] monohydrate] Geometric parameters (Å, º)

**Table:**

I1—Cu1^i^	2.7063 (13)	C3—C2	1.339 (11)
I1—Cu1	2.7216 (13)	C3—N1	1.381 (10)
I2—Cu1	2.6225 (12)	C2—H2	0.9300
I3—Cu1	2.6433 (11)	C2—N2	1.383 (12)
C1—H1	0.9300	O3—H3D	0.8634
C1—N1	1.303 (11)	O3—H3E	0.8224
C1—N2	1.303 (11)	O2—C6	1.202 (12)
C5—H5	0.9800	O1—H1A	0.8200
C5—C4	1.542 (13)	O1—C6	1.308 (12)
C5—N3	1.498 (11)	N3—H3A	0.8900
C5—C6	1.527 (11)	N3—H3B	0.8900
C4—H4A	0.9700	N3—H3C	0.8900
C4—H4B	0.9700	N1—H1B	0.8600
C4—C3	1.511 (12)	N2—H2A	0.8600
			
Cu1^i^—I1—Cu1	125.80 (3)	C2—C3—N1	105.7 (7)
I1^ii^—Cu1—I1	109.20 (4)	N1—C3—C4	121.6 (7)
I2—Cu1—I1	107.70 (4)	C3—C2—H2	126.6
I2—Cu1—I1^ii^	109.68 (5)	C3—C2—N2	106.8 (7)
I2—Cu1—I3	106.19 (4)	N2—C2—H2	126.6
I3—Cu1—I1^ii^	110.75 (4)	H3D—O3—H3E	103.6
I3—Cu1—I1	113.21 (5)	C6—O1—H1A	109.5
N1—C1—H1	125.8	C5—N3—H3A	109.5
N2—C1—H1	125.8	C5—N3—H3B	109.5
N2—C1—N1	108.3 (8)	C5—N3—H3C	109.5
C4—C5—H5	107.8	H3A—N3—H3B	109.5
N3—C5—H5	107.8	H3A—N3—H3C	109.5
N3—C5—C4	111.2 (7)	H3B—N3—H3C	109.5
N3—C5—C6	108.4 (7)	O2—C6—C5	122.6 (8)
C6—C5—H5	107.8	O2—C6—O1	125.9 (8)
C6—C5—C4	113.7 (7)	O1—C6—C5	111.5 (8)
C5—C4—H4A	108.3	C1—N1—C3	110.0 (7)
C5—C4—H4B	108.3	C1—N1—H1B	125.0
H4A—C4—H4B	107.4	C3—N1—H1B	125.0
C3—C4—C5	116.0 (7)	C1—N2—C2	109.2 (7)
C3—C4—H4A	108.3	C1—N2—H2A	125.4
C3—C4—H4B	108.3	C2—N2—H2A	125.4
C2—C3—C4	132.6 (8)		
			
C5—C4—C3—C2	73.1 (12)	N3—C5—C4—C3	63.6 (9)
C5—C4—C3—N1	−111.8 (9)	N3—C5—C6—O2	−0.1 (11)
C4—C5—C6—O2	124.1 (9)	N3—C5—C6—O1	−179.7 (8)
C4—C5—C6—O1	−55.5 (10)	C6—C5—C4—C3	−59.0 (10)
C4—C3—C2—N2	176.1 (8)	N1—C1—N2—C2	0.4 (13)
C4—C3—N1—C1	−176.5 (8)	N1—C3—C2—N2	0.5 (10)
C3—C2—N2—C1	−0.6 (12)	N2—C1—N1—C3	−0.1 (12)
C2—C3—N1—C1	−0.3 (10)		

Symmetry codes: (i) −*x*, *y*−1/2, −*z*+1; (ii) −*x*, *y*+1/2, −*z*+1.

### catena-Poly[4-[(2S)-2-azaniumyl-2-carboxyethyl]-1H-imidazol-3-ium [[diiodidocuprate(I)]-µ-iodido] monohydrate] Hydrogen-bond geometry (Å, º)

**Table:**

*D*—H···*A*	*D*—H	H···*A*	*D*···*A*	*D*—H···*A*
O3—H3*D*···I3^iii^	0.86	3.02	3.768 (8)	146
O3—H3*E*···I2	0.82	3.12	3.938 (7)	177
O1—H1*A*···O3^iv^	0.82	1.81	2.605 (10)	162
N3—H3*A*···I3^ii^	0.89	2.69	3.571 (8)	169
N3—H3*B*···I2^v^	0.89	2.75	3.601 (8)	160
N3—H3*C*···I2	0.89	2.79	3.596 (7)	152
N1—H1*B*···I1^ii^	0.86	2.81	3.516 (7)	141
N2—H2*A*···O2^vi^	0.86	1.97	2.804 (9)	163
C4—H4*A*···I2^vii^	0.97	2.88	3.716 (9)	146

Symmetry codes: (ii) −*x*, *y*+1/2, −*z*+1; (iii) −*x*+1, *y*+1/2, −*z*+2; (iv) *x*, *y*, *z*−1; (v) −*x*+1, *y*+1/2, −*z*+1; (vi) −*x*, *y*−1/2, −*z*; (vii) −*x*+1, *y*−1/2, −*z*+1.
